# Predicting six month follow‐up of suicidal thoughts and attempts among youth with anxiety presenting to an emergency department

**DOI:** 10.1002/jcv2.70096

**Published:** 2026-01-22

**Authors:** Marianne G. Chirica, Lauren O’Reilly, Cheryl A. King, Steven A. Miller

**Affiliations:** ^1^ Department of Psychological and Brain Sciences Indiana University Bloomington Bloomington Indiana USA; ^2^ Department of Psychiatry Indiana School of Medicine Indianapolis Indiana USA; ^3^ Department of Psychiatry University of Michigan Ann Arbor Michigan USA; ^4^ Department of Psychology Rosalind Franklin University of Medicine and Science North Chicago Illinois USA

**Keywords:** anxiety, depression, hopelessness, suicidal ideation, suicide attempts, youth

## Abstract

**Background:**

Previous research has established an association between anxiety disorders and suicidal thoughts and attempts; however, much remains unknown about the role of specific anxiety symptoms, anxiety severity, and the impact of co‐occurring depression and hopelessness. This study examined (a) the independent relationship between anxiety severity and suicidal thoughts and attempts, and (b) the independent relationships between different anxiety symptoms and suicidal thoughts and attempts. Additionally, we analyzed a subset of youth with anxiety symptoms to examine, (c) depression severity and hopelessness as predictors of suicidal thoughts and attempts.

**Methods:**

Participants were 2104 youth (ages 12–17) who presented to an Emergency Department (ED) from the Emergency Department Screen for Teens at Risk for Suicide (ED‐STARS) cohort. Youth self‐reported anxiety, depression, and hopelessness at baseline and reported suicidal thoughts and attempt at three‐ and/or 6‐month follow‐up. Among the subset of 652 youth with anxiety (≥3 on SCARED‐C), depression and hopelessness were examined as predictors of suicidal thoughts and attempts.

**Results:**

Anxiety severity significantly predicted ideation at follow‐up, even after accounting for demographics (race/ethnicity, sex, parental education, welfare), depression, hopelessness, and previous suicide attempts (OR = 1.08, 95% CI [1.01–1.61]). However, anxiety severity did not predict suicide attempts after accounting for depression. Specific anxiety domains were not associated with attempts, and only separation anxiety was associated with ideation (OR = 1.04, 95% CI [1.00–1.08]). Among the subset of youth reporting anxiety, depression severity was associated with suicide attempts at follow‐up, (OR = 1.08, 95% CI [1.04–1.14]). Depression (OR = 1.11, 95% CI [1.08–1.15]) and hopelessness (OR = 1.03, 95% CI [1.01–1.05]) each uniquely predicted suicidal ideation.

**Conclusion:**

Anxiety severity, rather than specific anxiety domains, may drive subsequent suicidal thoughts and attempts. Among youth with anxiety, depression predicted both attempts and ideation at follow‐up, whereas hopelessness predicted only ideation. Shared aspects of anxiety and depression may underlie youth suicide risk.

## Key points

**What's known?**

- Anxiety disorders are highly prevalent among youth, with lifetime prevalence estimates as high as 44%.
- Youth anxiety has been associated with suicidal thoughts and behaviors, though this relationship is often complicated by comorbid conditions such as depression and hopelessness.

**What's new?**

- This study clarifies the independent predictive role of anxiety by accounting for key comorbidity and confounding factors.
- Greater anxiety severity predicts suicidal ideation at three‐ and/or 6‐month follow‐up, even after adjusting for prior suicidal thoughts and attempts, depression, hopelessness, and demographic variables.
- Among youth with anxiety, depression and hopelessness both uniquely predict suicidal ideation, but only depression significantly predicts attempts.

**What's relevant?**

- Findings underscore the importance of comprehensive psychopathology screening in emergency department settings.
- Screening approaches should assess anxiety, depression, and hopelessness (as opposed to depression alone) in ED settings, where many youths present in the months prior to a suicide attempt.

## INTRODUCTION

Anxiety disorders are a group of psychiatric disorders characterized by various fears that cause significant distress or impairment. Although anxiety disorders share characteristics, they can be differentiated by the feared object or situation (Ramsawh et al., 2010). Anxiety disorders are a prevalent problem in youth, with a reported lifetime prevalence as high as 44% (Parodi et al., 2022), compared to the lifetime prevalence of depression of 19% (Shorey et al., 2022). Symptoms across anxiety disorders are similar, and have comorbidity with other diagnoses (Essau & de la Torre‐Luque, 2023; Kalin, 2020), such as depression (20%–70% lifetime prevalence; Kalin, 2020) and substance use disorders, which have a 16.5% 12‐month prevalence (Grant et al., 2004; Kalin, 2020). Moreover, anxiety disorders among adolescents may be associated with severe functional impairment, reduced school attendance, and poor social functioning (Mian et al., 2020; Ramsawh et al., 2012).

The few existing prospective meta‐analyses, spanning all ages, indicate that anxiety disorders predict suicidal thoughts and attempts (Bentley et al., 2016; Kanwar et al., 2013). Suicide was the second leading cause of death among youth aged 10–14 and the third leading cause of death for adolescents and young adults aged 15–24 in the United States (U.S.) from 2018 to 2022 (Centers for Disease Control and Prevention & National Center for Injury Prevention and Control, 2024). With such a high prevalence for youth anxiety disorders, it is crucial to study the relationship between youth anxiety and suicidal thoughts and attempts.

There are several key criteria for examining this relationship to adequately establish anxiety as a risk factor for suicidal thoughts and attempts (Hill et al., 2011). Anxiety must first be significantly associated with suicidal thoughts and attempts. Second, the association must not be caused by a third variable or set of variables. Third, anxiety must temporally precede suicidal thoughts and attempts.

### Association between anxiety and suicidal thoughts and attempts

In adults, anxiety disorders have been significantly associated with suicidal ideation and attempts (Nepon et al., 2010; Sareen et al., 2005). History of suicide attempts is also more frequent in adults with anxiety disorders than in the general population (De La Vega et al., 2018). Fewer studies have examined these relationships in children and adolescents, although King et al. (2019) reported associations between mean anxiety and suicide attempts in youth, unadjusted odds ratio (OR) = 1.2; (95% CI, 1.1, 1.2). A comprehensive review of all anxiety disorders in youth (Hill et al., 2011) concluded that although there is evidence of a significant association between anxiety and suicidal thoughts and attempts in youth, it is unclear whether the association with anxiety is due to a third variable. Furthermore, it is unclear whether anxiety temporally precedes suicidal thoughts and attempts because most literature is cross‐sectional.

Studies examining the risk of specific anxiety disorders, rather than presence of any anxiety disorder, in youth are scant and results have been inconclusive. For example, a comprehensive meta‐analysis (Leigh et al., 2023) found a significant cross‐sectional association between social anxiety and suicidal ideation and attempts in youth; however, due to a lack of longitudinal studies, prospective associations were not examined. Moreso, a review of adults found suicide risk and suicide rates are higher in patients with *any* anxiety disorder than those without anxiety, except for obsessive‐compulsive disorder (Kanwar et al., 2013). Other studies report an elevated risk of death by suicide among individuals with obsessive‐compulsive disorder (de la Cruz et al., 2024; Sidorchuk et al., 2021). More research is needed to identify if different anxiety disorders and severity of anxiety (i.e., total anxiety symptoms) are differentially associated with subsequent suicidal thoughts and attempts. Research in adults supposes that anxiety severity or anxiety comorbidity may be the driving factor for resulting suicidal thoughts and attempts rather than specific disorders (Coryell et al., 2019), yet this association has yet to be distinguished in youth.

### Accounting for potential confounding variables

Given the comorbidity among anxiety diagnoses in youth (Kalin, 2021) and their overlap with depression (Kessler et al., 2005), it is essential to account for potential confounding due to shared variance between anxiety, depression, and hopelessness. Two potential confounding variables are considered: (1) depression and (2) hopelessness. Depression is particularly relevant given its frequent co‐occurrence with anxiety (Groen et al., 2020) and its role in the tripartite model of anxiety and depression (Clark & Watson, 1991). This model posits that general distress underlies both disorders, offering a theoretical rationale for their co‐occurrence (Joiner Jr et al., 1999; Miller & Grotkowski, 2018).

Commonly established risk factors for suicidal thoughts and attempts include prior suicide attempts and psychiatric diagnoses, particularly depression (King et al., 2019). Suicide risk is further heightened among individuals with comorbid anxiety and mood disorders (De La Vega et al., 2018), a pattern frequently observed in youth (Groen et al., 2020). Anxiety and depression demonstrate a bidirectional relationship: some studies suggest that anxiety disorders precede and predict later depression (Cummings et al., 2014; Mathew et al., 2011), while others indicate the reverse—that depression may increase the likelihood of subsequent anxiety (Hamilton et al., 2016). Given these overlapping pathways, depression can be conceptualized as a confounding variable in the association between anxiety and suicidal thoughts and attempts, underscoring the importance of accounting for it in research.

Many studies reviewed by Hill et al. (2011) did not address depressive symptoms or diagnoses. Among those that did, findings were mixed. Some studies identified a persistent association between panic symptoms and suicidal thoughts and attempts even after controlling for depression (Pilowsky et al., 2000; Valentiner et al., 2002), whereas others found an attenuated association between anxiety and suicidal thoughts and attempts when depression was included in the model (Foley et al., 2006; Lewinsohn et al., 1996; Thompson et al., 2005). For suicidal ideation specifically, evidence is similarly inconsistent. Several studies found an association between anxiety symptoms and ideation that remained significant after controlling for depression scores (Carter et al., 2008; Ghaziuddin et al., 2000; Valentiner et al., 2002), whereas others did not (Esposito & Clum, 2002; Greene et al., 2009; Prinstein et al., 2000). These inconsistencies highlight the need for careful conceptualization of depression within the causal pathway linking anxiety and suicidal thoughts and attempts. Without adequately controlling for depression, the strength of the association may be overestimated, reflecting shared variance rather than a distinct contribution of anxiety.

Hopelessness, defined as negative expectations about the future (Beck et al., 1974), also has been consistently linked to and predictive of suicidal thoughts and attempts (Beck et al., 1985). Hopelessness is often examined alongside depression as a suicide risk factor, consistent with major theories that view hopelessness as neither necessary (e.g., Interpersonal‐Psychological Theory of Suicide (Joiner, 2005); nor sufficient (e.g., Hopelessness Theory of Suicide Joiner et al., 2002); to cause suicidal behavior. Hopelessness also contributes as a core symptom of depression, further complicating their distinction.

Longitudinal findings on hopelessness and depression as predictors of suicidal outcomes in adolescents are limited but suggestive. In adolescents with depression, hopelessness predicted suicidal ideation over 6 weeks (Wolfe et al., 2019), and was a stronger long‐term predictor of repeated suicide attempts than depression among youth with prior attempts (Groholt et al., 2006). Further research is needed to clarify the relative contributions of depression and hopelessness to suicide risk across developmental stages.

### Temporal precedence of anxiety relative to suicidal outcomes

Few studies have taken prospective, longitudinal approaches to examining the relationship between anxiety and suicidal thoughts and attempts in youth (Goldston et al., 1999; King et al., 2019; Weissman et al., 1999; Wolk & Weissman, 1996); Most have relied on cross‐sectional designs, limiting the ability to establish anxiety as a risk factor. Establishing temporal precedence is needed to make conclusions about the predictiveness of factors for suicidal thoughts and attempts. Adjusting for past suicidal thoughts and attempts is pertinent when studying predictive factors for future suicidal thoughts and attempts because past suicidal thoughts and attempts is predictive of future suicidal attempts. Thus, there is a clear need for longitudinal research examining the association between anxiety and suicidal thoughts and attempts in youth. King et al. (2019) accounted for previous suicidal thoughts and attempts in multi‐variable models and did not find youth anxiety to be predictive of suicidal attempts. It is important to further expand on this research because there are other precursors to suicidal thoughts and attempts, such as depression and hopelessness that could impact this relationship as well as suicidal ideation.

### Current study

Given limitations in prior research, our first aim was to identify the independent association between anxiety severity (total anxiety symptom score) and suicidal thoughts and attempts at three‐ and/or 6‐month follow‐up among youth presenting to pediatric emergency departments. Building on previous univariate findings (King et al., 2019), hierarchical regressions sequentially adjusted for potential confounders (demographics, depression severity, hopelessness, and prior suicide attempts) to test for third‐variable influences. Our second aim assessed the relationship between specific anxiety domains and suicidal thoughts and attempts at follow‐up, controlling for the same covariates. The third aim focused on a subset of youth with anxiety (*n* = 652; 63% of the total sample) to evaluate how depression severity and hopelessness predict suicidal thoughts and attempts. We hypothesized that anxiety symptoms would predict suicidal thoughts and attempts, with some variance shared with depression.

## METHODS

### Participants and procedures

Adolescents (ages 12–17) were recruited from 13 Emergency Departments (ED) in collaboration with the Pediatric Emergency Care Applied Research (PECARN; June 2015–July 2016); 10,664 adolescents were approached, of whom 6549 (61.4%) completed a suicide risk survey. A subset of patients (*n* = 2850 (43.6%)) were randomly assigned to a three and 6‐month telephone follow‐up; 2,104 participants completed this follow‐up (72% retention). Because participants at moderate to high suicide risk were preferentially selected for follow‐up, the resulting sample was enriched for suicide risk and not fully representative of the baseline cohort (King et al., 2021). Details of the randomization procedure and enrichment algorithm are provided elsewhere (King et al., 2019). The follow‐up sample was primarily female (63.1%) *M*_age_ = 15.1, SD = 1.6.

**FIGURE 1:**
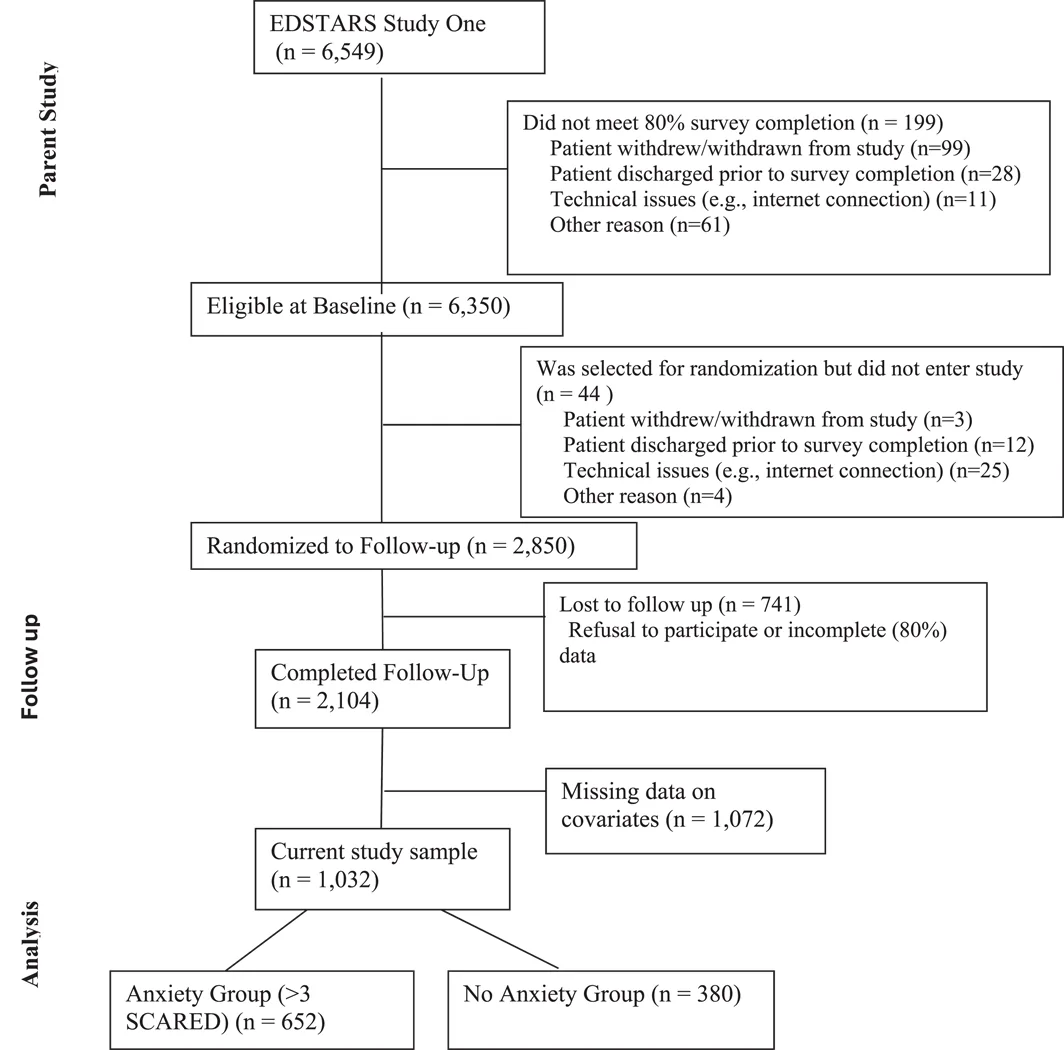
Flowchart of participants.

Adolescents were recruited during randomly selected screening shifts at sites during time periods when research coordinators were on‐site. Exclusion criteria included: previous study enrollment, being a ward of the State, non‐English speaking adolescents and non‐English speaking parents, medically unstable and/or severe cognitive impairment. Adolescents answered self‐report items about demographics and suicide risk factors in a survey while in the ED. Participants were included if adolescent and parent (*n* = 1,799, 85.5%), adolescent only (*n* = 183, 8.7%), or parent only (*n* = 122, 5.8%) conducted the follow‐up interviews. Participants were also only included if they completed over 80% of the assessments at baseline. All parent/guardians and adolescents provided written‐informed consent/assent. Participants were remunerated with online gift certificates. This study was exempt from Institutional Review Board (IRB) approval because it relied exclusively on de‐identified data from a public‐use dataset and therefore did not involve human subjects research. See Figure 1 For a participant flowchart.

### Measures

This study used youth‐self report survey data from baseline (92 primary questions) and follow‐up (27 questions) of EDSTARS Study 1. See Appendix S1 for details.

#### Baseline assessment of primary study variables

##### Anxiety

Anxiety experienced in the past 3 months was assessed with the Screen for Child Anxiety Related Emotional Disorders (SCARED‐C (Short form)) (Birmaher et al., 1999; Canals et al., 2012). The five items of the SCARED‐C represent specific anxiety disorders found in youth: “I get really frightened for no reason at all”‐ panic disorder/somatic symptoms; “I am shy”‐ social anxiety; “I am scared to go to school”‐ school avoidance; “People tell me that I worry too much”‐ generalized anxiety; and “I am afraid to be alone in the house”‐ separation anxiety. Birmaher et al. (1999) found reliability and validity evidence for this scale. A hierarchical omega was computed on the SCARED‐C to determine amount of unique variance associated with each domain and ensure sufficient unique variance to proceed with domain‐specific regressions. Preliminary analyses revealed a hierarchical McDonald's omega of 0.64 and a Cronbach's alpha of 0.64, indicating that individual SCARED‐C items shared moderate variance on a common higher‐order factor while also capturing unique aspects of specific symptoms. Consistent with previous literature using the SCARED‐C short form, a cutoff score greater than 3 was used to identify youth at risk for clinical anxiety when examining anxiety severity (Birmaher et al., 1999; Canals et al., 2012).

##### Hopelessness

Hopelessness was assessed by the question, “I thought there was nothing good for me in the future.”

##### Depression

Depression was measured by The Patient Health Questionnaire (PHQ‐9) total score (Kroenke & Spitzer, 2002). This commonly used self‐assessment for depression has been found valid and reliable (Kroenke et al., 2001). There are 9 symptom items with an additional rating of impairment. Each item response ranges from 0 (not at all) to 3 (nearly every day). The modified sum score (excluding the hopelessness item) demonstrated good internal consistency (*α* = 0.89).

##### Other items

Other baseline characteristics included as covariates included lifetime history of suicidal ideation and suicide attempts, recent suicidal ideation, sex, ethnicity/race and parent demographics (i.e., mother and father educational attainment and welfare benefit status). Baseline measures of lifetime suicidal ideation and attempts, recent suicidal ideation, and (NSSI) were used as covariates in the model to assess suicidal thoughts and attempt outcomes.

#### Primary study outcomes

Suicide attempts and suicidal ideation were assessed at the three‐ and 6‐month follow‐up assessments using the adapted Columbia–Suicide Severity Rating Scale (C‐SSRS; Posner et al., 2008). The C‐SSRS has been validated among diverse populations in both clinical and research settings (Posner et al., 2011; Reeves et al., 2022). Consistent with previous research (King et al., 2019), suicide attempt was defined as positive response to either C‐SSRS question: “In the past 3 months, have you made a suicide attempt?” “In the past 3 months, have you tried to harm yourself because you were at least partly trying to end your life?” Responses were combined into a single outcome to reflect if patients responded at either three‐ or 6‐month follow‐up.

Suicidal ideation was assessed using the C‐SSRS severity scale, which evaluates multiple characteristics of ideation: frequency, duration, controllability, deterrents, and reason for ideating (Reeves et al., 2022). Responses were combined into a categorical outcome to reflect if patients had ideation or did not have ideation at three‐ or 6‐month follow‐up. Participants who selected “unknown” or left these items blank were excluded from analyses to accurately distinguish youth with and without suicidal thoughts and attempts.

### Statistical analysis

All analyses were conducted in R version 4.3.1. Continuous variables were examined for normality, and missing data were handled via listwise deletion. Logistic regression models were used for binary outcomes. Analyses were conducted in four steps: (1) unadjusted models, (2) models adjusting for demographic variables, (3) models adjusting for demographic variables and baseline suicide‐related variables, and (4) fully adjusted models including depression severity and hopelessness. Statistical significance was determined at *p* < 0.05 (two‐tailed).

To assess the robustness of results, all models were re‐estimated with and without depression severity and hopelessness, as well as excluding baseline suicidal ideation and attempt history. Model assumptions, including multicollinearity and linearity of continuous variables, were evaluated and met prior to analysis.

#### Aim 1: Independent associations between anxiety severity and suicidal ideation and attempts

Univariate regression models examined associations between overall anxiety severity and suicidal thoughts and attempts at follow‐up. Models were progressively adjusted in three steps: (1) Demographic covariates (sex, race/ethnicity, and parent education); (2) baseline suicide‐related variables (lifetime attempt history, lifetime suicidal ideation, and recent suicidal ideation); and (3) depression severity and hopelessness.

#### Aim 2: Independent associations between specific anxiety domains and suicidal thoughts and attempts

Multiple regression models were estimated with all five anxiety domains (generalized anxiety, social anxiety, school avoidance, separation anxiety, and panic/somatic symptoms) entered simultaneously to examine their unique associations with suicidal thoughts and attempts. The same three‐step covariate adjustment approach described above was applied.

#### Aim 3: Predictiveness of depression and hopelessness among youth with anxiety

Among a subsample of youth with elevated anxiety (*n* = 652), logistic regression models compared the relative predictive value of depression severity and hopelessness for suicide attempt and ideation outcomes at follow‐up. Full and reduced model comparisons were conducted using likelihood ratio (*χ* ^2^) and *F*‐tests to evaluate incremental predictive utility.

## RESULTS

### Specific study sample

Of 6549 adolescents from the ED‐STARS Study One, 6109 (96.9%) met baseline survey completion criteria (≥80%). Of these, 1032 had complete data on all variables and covariates and were included in the present analyses. The Anxiety Group (SCARED‐C ≥3) included 652 youth (63% of the total sample), while the Non‐Anxiety Group (SCARED‐C <3) included 380 youth. Figure 1 illustrates participant retention. A total of 1083 youth provided follow‐up data on suicidal thoughts and attempts, forming the analytic sample for predictive analyses.

### Descriptive statistics

At baseline, youth displayed relatively low but variable levels of depression severity (Mean = 6.31, SD = 6.52, Range = 0–27). Table 1 presents detailed baseline demographics and characteristics by anxiety group.

**TABLE 1:** Demographics of youth divided by anxiety severity (*N* = 1032).

Sample characteristics	Anxiety group (≥3) (*n* = 652)	No anxiety group (*n* = 380)
Birth sex: Female	511 (78.4%)	220 (57.9%)
Race		
American Indian or Alaska Native	23 (3.5%)	15 (3.9%)
Asian/Native Hawaiian/Pacific Islander	10 (1.5%)	6 (1.6%)
Black or African American	152 (23.3%)	122 (32.1%)
Latinx	6 (0.9%)	4 (1.1%)
White	474 (72.7%)	242 (63.7%)
Unknown/unavailable	26 (8.1%)	19 (5.0%)
Childs grade in school		
5th–8th grade	177 (27.1%)	138 (36.3%)
9th‐high school graduate	471 (72.2%)	241 (63.4%)
Child does not attend school	4 (0.6%)	1 (0.3%)
Mother/Stepmother education		
High school graduate or less	152 (23.3%)	95 (25.0%)
Some college/technical training	213 (32.7%)	116 (30.5%)
College graduate/professional	281 (43.1.0%)	164 (43.2%)
Don't know/Not applicable	6 (0.9%)	5 (1.3%)
Father/Stepfather education		
High school graduate or less	228 (35.0%)	150 (39.5%)
Some college/technical training	147 (22.5%)	79 (20.8%)
College graduate/professional	228 (34.9%)	121 (31.8%)
Don't know/Not applicable	49 (7.5%)	30 (7.9%)
Family public Assistance	248 (38.0%)	165 (43.4%)

#### Aim 1: Associations between overall anxiety severity and suicidal thoughts and attempts

Logistic regressions models using the SCARED‐C total score, adjusted for demographic covariates, indicated that anxiety severity was significantly associated with suicide attempts at follow‐up (OR = 1.29, 95% CI [1.19–1.39]). This corresponds to a 29% increase in odds of a suicide attempt for each unit increase in anxiety symptoms. After adjusting for baseline suicidal ideation and past suicide attempts, the association was attenuated and no longer significant (OR = 1.08, 95% CI [0.99–1.19]).

In logistic regression models predicting suicidal ideation, anxiety severity remained significantly associated with ideation after adjusting for baseline covariates, depression, and hopelessness (OR = 1.08, 95% CI [1.01–1.61]). Table 2 reports all estimates between anxiety severity and suicidal thoughts and attempts.

**TABLE 2:** Anxiety severity predicting suicidal thoughts and attempts.

	Suicide attempts		Suicidal ideation	
	OR	95% CI	OR	95% CI
Independent association	1.21	1.12–1.31	1.29	1.22–1.37
+Demographics^a^	1.29	1.19–1.39	1.20	1.10–1.30
+Previous suicidal behavior^b^	1.08	0.99–1.19	1.15	1.08–1.23
+Depression, hopelessness	1.05	0.95–1.16	1.08	1.01–1.61
+Depression, hopelessness but no previous suicide attempts	1.07	0.97–1.18	1.09	1.02–1.17

^a^ Demographic covariates include gender, ethnicity, parental education level (father and mother), and welfare.

^b^ Previous suicidal behavior includes lifetime suicidal ideation, lifetime history of a suicide attempts, and recent suicidal ideation.

#### Aim 2: Associations between specific anxiety domains and suicidal thoughts and attempts

Multiple regression models simultaneously including all five anxiety domains were estimated to examine their unique associations with suicidal thoughts and attempts. For suicidal ideation, the only significant associations were observed for separation anxiety (OR = 1.04, 95% CI [1.00–1.08]). No anxiety domains were significantly associated with suicide attempts. Table 3 provides full estimates between specific anxiety domains and suicidal thoughts and attempts.

**TABLE 3:** Specific anxiety disorders predicting suicidal thoughts and attempts.

	Suicide attempts		Suicidal ideation	
	OR	95% CI	OR	95% CI
Social anxiety	1.04	0.75–1.44	1.00	0.97–1.04
Generalized anxiety disorder	1.03	0.75–1.42	0.99	0.96–1.03
School avoidance	0.97	0.68–1.37	1.04	0.99–1.09
Panic disorder/somatic symptoms	1.17	0.83–1.66	1.11	0.97–1.06
Separation anxiety	1.21	0.90–1.61	1.04	1.00–1.08

*Note*: *Includes all covariates.

#### Aim 3: Associations of depression and hopelessness among youth with anxiety

Among youth with anxiety (*n* = 652), depression severity was significantly associated with suicide attempts at follow‐up (OR = 1.08, 95% CI [1.04–1.14]), while hopelessness was not (OR = 1.01, 95% CI [0.99–1.04]). Each one unit increase in depression severity corresponded to an 8% increase in the odds of a suicide attempt.

For suicidal ideation, both depression severity (OR = 1.11, 95% CI [1.08–1.15]) and hopelessness (OR = 1.03, 95% CI [1.01–1.05]) were uniquely associated with ideation, with depression showing a stronger association. A model comparison using an *F*‐test indicated a significant difference in predictiveness (F (1,648) = 9.76, *p* < 0.001). Each one‐unit increase in depression severity corresponded to a 2% increase in suicidal ideation, and each one‐unit increase in hopelessness corresponded to a 0.7% increase. Together, depression and hopelessness accounted for 16% of the variance in suicidal ideation. Table 4 presents full estimates.

**TABLE 4:** The predictiveness of depression and hopelessness for suicide attempts and suicidal ideation among youth with anxiety.

	Suicide attempts		Suicidal ideation	
	OR	95% CI	OR	95% CI
Depression	1.08	1.04–1.14	1.11	1.08–1.15
Hopelessness	1.01	0.99–1.04	1.03	1.01–1.05

## DISCUSSION

### Overview

This study identified the independent association between anxiety (i.e., anxiety severity and specific anxiety symptoms) and suicidal thoughts and attempts at three‐ and/or 6‐month follow‐up among youth presenting to pediatric emergency departments. Our study tested the relationship between anxiety severity and anxiety domains and subsequent suicidal thoughts and attempts in youth, while adjusting for potential third‐variable influences, such as depression severity and hopelessness. Consistent with previous theories of anxiety, depression, and suicide, we examined the extent to which depression severity and hopelessness predicted suicidal thoughts and attempts in a subsample of youth with anxiety, with findings revealing depression severity as a significant predictor in the relationship between anxiety and suicide attempts. Hopelessness also linearly predicted the relationship between anxiety and suicidal ideation. Additionally, we established temporal precedence while testing our association between anxiety and suicidal thoughts and attempts by controlling for previous suicide attempts and ideation.

### Findings

Among all youth, anxiety severity significantly predicted suicide attempts when controlling for demographics, but this significant relationship attenuated when previous suicide attempts, suicidal ideation, depression severity and hopelessness were included in the model. Consistent with King et al. (2019), this association first became attenuated when previous suicidal thoughts and attempts were accounted for. Results are consistent with previous studies examining anxiety and suicidal thoughts and attempts broadly, finding attenuation in relationships after controlling for depression severity. Youth with anxiety are at higher risk for suicide attempts; this relationship is partially explained by previous suicidal thoughts and attempts, depression, and hopelessness.

Contrary to some past research (Esposito & Clum, 2002; Greene et al., 2009; Prinstein et al., 2000), but in line with other research (Carter et al., 2008; Ghaziuddin et al., 2000; Valentiner et al., 2002), our results indicate that anxiety severity significantly predicts follow‐up suicidal ideation, even after controlling for all covariates. Notably, anxiety remains a significant risk factor above and beyond depression severity, hopelessness, and prior suicidal attempts or ideation, highlighting its unique contribution to suicide risk in youth. As Carter et al. (2008) emphasize, these findings may be influenced by the type of anxiety measure employed; trait versus state anxiety assessments, as well as differences in sample characteristics, could account for discrepancies across studies. Our results underscore the importance of assessing anxiety directly, rather than only in the context of depression, when evaluating suicide risk.

When evaluating individual anxiety symptom domains as predictors of suicidal thoughts and attempts in youth, no domains independently predicted suicide attempts. In contrast, separation anxiety emerged as the only domain significantly associated with suicidal ideation. Previous research in this sample also found univariate associations between anxiety severity with suicide attempts (King et al., 2019), supporting our findings. For both suicidal thoughts and attempts, our findings differ from prior studies that reported associations between panic/anxiety symptoms and suicidal thoughts or attempts after controlling for depression (Pilowsky et al., 2000; Valentiner et al., 2002). Several factors may account for these discrepancies. First, differences in sample characteristics, such as age range, clinical versus community samples, or baseline risk, may influence observed associations. Second, differences in the measurement of anxiety symptoms or suicidal outcomes, including reliance on self‐report and single‐item indicators for specific anxiety domains, may have influenced the results. Third, differences in statistical modeling, including the simultaneous inclusion of multiple anxiety domains and covariates, may attenuate the unique contribution of panic/anxiety symptoms. These factors suggest that associations between specific anxiety symptoms and suicidal outcomes may be context‐dependent and warrant further investigation.

Despite the Cognitive Theory of Depression suggesting hopelessness (defined by a negative outlook of the future) is a symptom of depression (Beck, 1979), our results found a significant difference between depression and hopelessness in their predictiveness of suicidal thoughts and attempts (indicating unique predictive ability in the overall variance). Among the subset of youth reporting anxiety, depression was predictive of suicide attempts, whereas hopelessness did not reveal a significant predictive association. This differs from other research which found the opposite; hopelessness, but not depression, has been found to predict suicide across a 13‐year period (Kuo et al., 2004). One possible explanation for this discrepancy is that our study removed hopelessness items from the depression measure to reduce criterion contamination, allowing depression and hopelessness to be examined as distinct constructs. Previous research, which may have included hopelessness items within depression scales, could have conflated these constructs, potentially leading to findings where hopelessness, but not depression, predicted suicidal outcomes.

Both depression severity and hopelessness predicted suicidal ideation, with each contributing unique variance. In this sample, depression severity accounted for more variance and emerged as the stronger predictor, suggesting that while hopelessness captures additional risk, depressive symptoms play a more central role in suicidal thoughts among youth with anxiety. These findings contrast with prior research identifying hopelessness as the stronger predictor (Groholt et al., 2006), likely reflecting sample or methodological differences. Together, depression and hopelessness explained 16% of the variance, indicating that shared features of anxiety and depression contribute to suicidal ideation. Factors such as the heightened salience or variability of depressive symptoms in an ED sample, broader measurement of depression, and prior conflation of depression and hopelessness may account for these differences.

### Strengths and limitations

This study had several considerable strengths, including its relatively large and diverse sample of adolescents, its prospective design with a follow‐up allowing for risk prediction three‐ and 6‐months after presentation in an ED, and oversampling for high‐risk youth. Additionally, this was a longitudinal study which allowed for temporal precedence. Despite these notable strengths, findings should be interpreted within the context of study limitations.

Although geographically diverse, our sample was not nationally representative, which may limit the generalizability of study findings. Additionally, adolescents were enrolled from pediatric EDs in academic health centers in urban areas, which may not be representative of the patient population found in EDs in other geographic and clinical settings. In terms of measurement, constructs of interest were measured with brief scales, however each of these scales have demonstrated predictive validity for suicide attempts (King et al., 2019). In particular, anxiety domains were assessed with one individual question from the SCARED‐C short‐form, so findings regarding the specific domains must be considered with caution. Additionally, all questions needed to be answered during the initial survey, and the exclusion of individuals with some degree of missing data may have introduced a potential source of bias for the final sample. Lastly, the use of a single‐item measure of hopelessness may account for differences between the present findings and prior literature. Future research employing multi‐item assessments of depression and anxiety to model latent constructs could provide a more nuanced understanding of how these factors relate to suicidal thoughts and behaviors.

Taken together, this study was able to establish a significant relationship between anxiety severity and suicidal ideation in youth, as well as establish separation anxiety as a potentially important predictor of suicidal ideation. Our findings support previous research that found an association between anxiety and suicidal ideation in youth that remained significant after controlling for dimensional depression scores (Carter et al., 2008; Ghaziuddin et al., 2000; Valentiner et al., 2002). Our study expands on the previous research by also controlling for previous suicidal thoughts and attempts, as well as hopelessness and demographics.

### Clinical implications

Screening youth for suicide risk is crucial from both clinical and public health perspectives to identify youth at risk and to prevent suicide attempts and deaths (Cwik et al., 2020). Our results indicate that anxiety severity predicts suicidal ideation over and above depression, hopelessness, and previous suicidal attempts and ideation, such that critical predictive information may be missed if youth are only screened for depression. Thus, more robust psychopathology screening that assays anxiety, depression, and hopelessness (as opposed to depression alone, e.g., the PHQ‐9) is needed in ED settings, where many youths present in the months prior to a suicide attempt (Cwik et al., 2020). For example, rapid, adaptive assessment of psychopathology could facilitate identification and referral of suicidal thoughts and attempts in ED settings (Gibbons et al., 2020). One such example is the Kiddie‐Computerized Adaptive Test (K‐CAT), a computerized adaptive test that assesses depression, anxiety, attention‐deficit/hyperactivity disorder, mania, substance use, psychosis, and overall suicide risk severity, among others in less than 2 mins per domain and yields dimensional severity assessment and measurement (Gibbons et al., 2020). Another example is the Computerized Adaptive Screen for Suicidal Youth (CASSY), which is a multi‐dimensional suicide risk screen requiring less than 2 mins for administration (King et al., 2021). These adaptive questionnaires can capture a variety of predictors for suicidal thoughts and attempts from multiple domains, while ultimately creating a score on a continuum. Without these supplementary screenings to the currently accepted depression screening, a wide range of youth with suicidal thoughts and attempts that present to an ED might be missed in screening and released without resources or intervention.

## CONCLUSION

We found that anxiety severity significantly predicted suicidal ideation when adjusting for all covariates. Anxiety severity significantly predicted suicide attempts when adjusting for demographics, but not previous suicidal ideation and attempts. Specific anxiety domains did not significantly predict suicide attempts, and only separation anxiety was significant. No individual anxiety domains were significant for suicide attempts. Overall, these results suggest that anxiety severity, rather than specific anxiety domains, may be the driving factor for resulting suicidal thoughts and attempts rather than specific disorders, consistent with previous research (Coryell et al., 2019).

Our study was able to test the relationship between anxiety (anxiety severity and specific anxiety domains) and subsequent suicidal thoughts and attempts in youth while adjusting for a potential third‐variable influence, such as depression and hopelessness. We were able to establish temporal precedence while testing our association by controlling for previous suicidal thoughts and attempts. These results have significant implications for youth suicide prevention as our findings can help tailor suicide screenings in youth.

## AUTHOR CONTRIBUTIONS

**Marianne G. Chirica**: Conceptualization; data curation; formal analysis; methodology; project administration; visualization; writing—original draft; writing—review and editing. **Lauren M O'Reilly**: Conceptualization; methodology; writing—review and editing; **Cheryl A. King**: Conceptualization; funding acquisition; investigation; project administration; resources; supervision; validation; writing—review and editing; **Steven A. Miller**: Conceptualization; data curation; formal analysis; methodology; software; supervision; visualization; writing—review and editing.

## CONFLICT OF INTEREST STATEMENT

The authors declare no conflicts of interest.

## ETHICAL CONSIDERATIONS

All parent/guardians and adolescents provided written‐informed consent/assent. Participants were remunerated with online certificates. This study was exempt from Institutional Review Board (IRB) approval because it relied exclusively on de‐identified data from a public‐use dataset and therefore did not involve human subjects research.

## Supporting information

Supporting Information S1

## Data Availability

The dataset and protocol are currently available to the public through the Pediatric Emergency Care Applied Research Network (PECARN) website at the following url: https://pecarn.org/datasets/.
