# Supplementary material for: Predicting six month follow‐up of suicidal thoughts and attempts among youth with anxiety presenting to an emergency department
Source: JCPP Adv. 2026 Jan 22:e70096. Online ahead of print. doi: 10.1002/jcv2.70096 (PMC13339218; doi:10.1002/jcv2.70096)
Supplement: Supplementary file 1 — Supporting Information S1 [file JCV2-9999-e70096-s001.docx]

**Predicting Six Month Follow-up of Suicidal Thoughts and Attempts among Youth with Anxiety Presenting to an Emergency Department**

**Supporting Information**

| Appendix S1.  BASELINE QUESTIONS | |  |
| --- | --- | --- |
| NAME | **LABEL** | |
| Sex | What sex were you born as? | |
| RACE Q | How would you describe your race? Select all that apply:  (choice=American Indian or Alaska Native) | |
| RACE Q | How would you describe your race? Select all that apply: (choice=Asian) | |
| RACE Q | How would you describe your race? Select all that apply: (choice=Black or African American) | |
| RACE Q | How would you describe your race? Select all that apply:  (choice=Native Hawaiian or Other Pacific Islander) | |
| RACE Q | How would you describe your race? Select all that apply: (choice=White) | |
| RACE Q | How would you describe your race? Select all that apply: (choice=Unknown) | |
| Ethnicity | What is your ethnicity? | |
| School grade | What grade are you in school? | |
| Mother education | How much school has your mother / stepmother completed? | |
| Father education | How much school has your father / stepfather completed? | |
| Welfare | Does your family currently receive public assistance (i.e., food stamps, Medicaid)? | |
| FCS | How much do people in your family understand you? | |
| FCS | How much does your family pay attention to you? | |
| SOCIAL | I have friends I'm really close to and trust completely. | |
| SOCIAL | Spending time with my friends is a big part of my life. | |
| SCHOOL | You feel close to people at your school. | |
| SCHOOL | You feel like you are part of your school. | |
| LES | Did the death of a close friend or family member occur in the last 3 months? | |
| LES | Did breaking up with a boyfriend / girlfriend occur in the last 3 months? | |
| LES | Were you suspended / expelled from school or arrested in the last 3 months? | |
| LES | Were your parents separated or divorced in the last 3 months? | |
| PDS | What sex were you born as? | |
| PDS | About the growth of body hair (body hair means underarm and pubic hair).  Would you say that your body hair has: | |
| PDS | Have you noticed a deepening of your voice? | |
| PDS | Have you begun to grow hair on your face? | |
| PDS | Have your breasts begun to grow? | |
| PDS | Have you begun to menstruate? | |
| ASQ | In the past few weeks, have you wished you were dead? | |
| ASQ | In the past few weeks, have you felt that you or your family would be better off if you were dead? | |
| ASQ | In the past week, have you been having thoughts about killing yourself? | |
| ASQ | Have you ever tried to kill yourself? | |
| CSSRS | Have you ever in your life wished you were dead or wished you could go to  sleep and not wake up? | |
| CSSRS | In the past month, have you wished you were dead or wished you could go to  sleep and not wake up? | |
| CSSRS | Have you ever in your life had any thoughts of killing yourself? | |
| CSSRS | In the past month, have you had any thoughts of killing yourself? | |
| CSSRS | In the past 24 hours, have you had any thoughts of killing yourself? | |
| CSSRS | Have you ever in your life thought about how you might kill yourself? | |
| CSSRS | In the past month, have you thought about how you might kill yourself? | |
| CSSRS | Have you ever in your life had these thoughts and had any intent to act on them? | |
| CSSRS | In the past month, have you had these thoughts and had any intent to act on them? | |
| CSSRS | Have you ever in your life started to work out or have you worked out the details of  how to kill yourself? | |
| CSSRS | In the past month, have you started to work out or have you worked out the details  of how to kill yourself? | |
| CSSRS | Did you ever intend to carry out this plan? | |
| CSSRS | In the past month, did you intend to carry out this plan? | |
| CSSRS | Have you ever in your life made a suicide attempt? Examples: swallowed any pills,  tried to shoot yourself, cut yourself with wish to die, hang yourself | |
| CSSRS | If YES, how many times in your life? | |
| CSSRS | In the past month, have you made a suicide attempt? Examples: swallowed any pills,  tried to shoot yourself, cut yourself with wish to die, hang yourself | |
| CSSRS | In the past 24 hours, have you made a suicide attempt? Examples: swallowed any pills,  tried to shoot yourself, cut yourself with wish to die, hang yourself | |
| CSSRS | Have you ever in your life tried to harm yourself because you were at least partly  trying to end your life? Examples: swallowed any pills, tried to shoot yourself,  cut yourself with wish to die, hang yourself | |
| CSSRS | If YES, how many times in your life? | |
| CSSRS | In the past month, have you tried to harm yourself because you were at least partly  trying to end your life? Examples: swallowed any pills, tried to shoot yourself,  cut yourself with wish to die, hang yourself | |
| CSSRS | In the past 24 hours, have you tried to harm yourself because you were at least partly  trying to end your life? Examples: swallowed any pills, tried to shoot yourself,  cut yourself with wish to die, hang yourself | |
| CSSRS | Have you ever in your life taken any steps toward making a suicide attempt or  preparing to kill yourself? Examples: saving pills, getting a gun, giving your things away,  writing a suicide note | |
| CSSRS | In the past month, have you taken any steps toward making a suicide attempt  or preparing to kill yourself? Examples: saving pills, getting a gun, giving your things away,  writing a suicide note | |
| CSSRS | Have you ever in your life started to do something to end your life but  someone or something stopped you before you did anything? | |
| CSSRS | In the past month, have you started to do something to end your life but  someone or something stopped you before you did anything? | |
| CSSRS | Have you ever in your life started to do something to end your life but  stopped yourself before you actually did anything? Examples: took out pills but  didn't swallow any, held a gun but changed your mind, went to the roof but didn't jump | |
| CSSRS | In the past month, have you started to do something to end your life but  stopped yourself before you actually did anything? Examples: took out pills but  didn't swallow any, held a gun but changed your mind, went to the roof but didn't jump | |
| SIC | When you have suicidal thoughts, how long do they last? | |
| SIC | How likely are you to act on your suicidal thoughts? | |
| SIC | When I have suicidal thoughts, it is hard to think about other things. | |
| SIC | My suicidal thoughts repeat over and over in my head. |  |
| SIC | When my suicidal thoughts persist, it is hard to resist acting on them. |  |
| NSSI | In the past 12 months, have you ever harmed or hurt your body on purpose,  such as cutting or burning your skin, or hitting yourself, without wanting to die? |  |
| PHQ | Feeling bad about yourself - or that you are a failure or have let yourself or your family down. |  |
| PHQ | Trouble concentrating on things, such as reading or watching television. |  |
| PHQ | Moving or speaking so slowly that other people could have noticed, or the opposite,  being so fidgety or restless that you have been moving around a lot more than usual. |  |
| PHQ | Thoughts that you would be better off dead or of hurting yourself in some way. |  |
| PHQ | Over the last two weeks, how hard has it been for you to do what you need to do and  get along with others? |  |
| HMFQ | I thought there was nothing good for me in the future. |  |
| IPAS | Over the past 6 months, have you had times when you became angry and enraged  with others in a way that was out-of-control or inappropriate? |  |
| IPAS | How many times has this happened in the past 3 months? |  |
| IPAS | During these times, have you verbally attacked someone? |  |
| IPAS | During these times, have you thrown things or destroyed objects? |  |
| IPAS | During these times, have you physically attacked someone? |  |
| UPPS | When I feel rejected, I will often say things that I wish I hadn't. |  |
| UPPS | It is hard for me to not act on my feelings. |  |
| UPPS | I often make matters worse because I act without thinking when I am upset. |  |
| UPPS | Sometimes I do impulsive things that I wish I hadn't. |  |
| YRBS | During the past 12 months, how many times were you in a physical fight? |  |
| YRBS | During your life, with how many people have you had sexual intercourse? |  |
| YRBS | During the past 30 days, did you take any diet pills, powders, or liquids without a doctor's advice,  or did you vomit or take laxatives to lose weight or to keep from gaining weight?  (Do not count meal replacement products such as Slim Fast.) |  |
| SCARED | I am shy. |  |
| SCARED | People tell me that I worry too much. |  |
| SCARED | I am scared to go to school. |  |
| SCARED | I get really frightened for no reason at all. |  |
| SCARED | I am afraid to be alone in the house. |  |
| PANAS | Joyful |  |
| PANAS | Cheerful |  |
| PANAS | Happy |  |
| PANAS | Lively |  |
| PANAS | Proud |  |
|  |  |  |
| FOLLOW UP (3 and 6 MONTHS AFTER BASELINE) | |  |
| PHQ | Hard to Get Along w/ Others |  |
| PHQ | Down, Depressed, or Hopeless |  |
| PHQ | Tired or Little Energy |  |
| PHQ | Poor Appetite or Overeating |  |
| PHQ | Thoughts of Hurting Self |  |
| PHQ | Trouble with Sleep | |
| PHQ | Trouble Concentrating | |
| PHQ | Moving Slow or Restless | |
| PHQ | Little Interest or Pleasure | |
| PHQ | Feeling Bad About Self | |
| HMFQ | Nothing Good in Future | |
| CSSRS - FU | Not Wake Up | |
| CSSRS - FU | Thoughts of Killing Self | |
| CSSRS - FU | How Might Kill Self | |
| CSSRS - FU | Any Intent to Act | |
| CSSRS - FU | Worked Out Details to Kill Self | |
| CSSRS - FU | Intend to Carry Out Plan | |
| CSSRS - FU | Suicide Attempt | |
| CSSRS - FU | Number of Suicide Attempts | |
| CSSRS - FU | Harm Self to End Life | |
| CSSRS - FU | Taken Steps Toward Suicide Attempt | |
| CSSRS - FU | Someone/Something Stopped Suicide Attempt | |
| CSSRS - FU | Stopped Self Before Ending | |
| NSSI | Num of Times Harmed Self Past Week | |
| MHSU | Ever Hospitalized | |
| MHSU | Visited ED | |
| MHSU | Taken Medication | |
| MHSU | Obtained Therapy/Counseling | |
